# Thermotherapy for shoulder pain: A protocol for systematic review

**DOI:** 10.1097/MD.0000000000028446

**Published:** 2022-01-21

**Authors:** Chen Bin, Shen Cimin, Na Li, Lu Wang, Dandan Chen

**Affiliations:** Department of Acupuncture and Moxibustion, Fenghua Hospital of Traditional Chinese Medicine, Ningbo, Zhejiang Province, China.

**Keywords:** protocol, shoulder pain, systematic review, thermotherapy

## Abstract

**Background:**

Shoulder pain is a common musculoskeletal disorder prompting many patients to seek treatment. Thermotherapy is a common treatment for shoulder which has been widely used in hospitals. But its efficiency has not been scientifically and methodically evaluated. This protocol aims to evaluate the efficacy and safety of thermotherapy for treating shoulder pain.

**Methods:**

Eight databases will be searched from their inception to October 2021. They are as follows: PubMed, Embase, Cochrane Library, ClinicalTrials.gov, China Knowledge Resource Integrated Database (CNKI), Weipu Database for Chinese Technical Periodicals (VIP), Chinese Biomedical Literature Database (CBM), and Wanfang Database. Two researchers will independently select studies, collect data, and assess the methodology quality by the Cochrane risk of bias tool.

**Results:**

The systematic review will provide high-quality evidence to assess the efficacy and safety of thermotherapy for shoulder pain as well as adverse events.

**Conclusion:**

The systematic review will provide evidence to assess the effectiveness and safety of thermotherapy therapy for shoulder pain patients.

**INPLASY registration number:**

INPLASY2021110086.

## 1 Introduction

In a US survey, musculoskeletal conditions were reported by 50% of adults.^[1]^ Indeed, a high prevalence of shoulder pain is experienced by over one-quarter of the general population in their lifetimes.^[2]^ In China, the incidence of women over 40 is 45%, and the incidence is 8% for urban population.^[3]^ Unlike more prevalent conditions such as low back pain, with a low risk of chronicity (i.e., persisting ≥12 weeks),^[4]^ nontraumatic musculoskeletal shoulder pain persists beyond 6 months in 50% of individuals.^[5]^ It is a disabling condition that negatively impacts quality of life, psychological well-being, and the ability to maintain employment.^[6]^

There are many related conservation treatment applied on shoulder pain, such as corticosteroids, nonsteroidal anti-inflammatory drugs, acupuncture, manual therapy, and other physical therapy. And the effectiveness of most intervention is not definite.^[7]^ Thermotherapy is the application of heat to the body resulting in increased tissue temperature,^[8]^ techniques for thermotherapy include the application of Moxibustion, hot packs, superficial heat, and via diathermy (application of electromagnetic energy). Thermotherapy is used in rehabilitation to reduce pain and stiffness, and to increase mobility.^[9]^ Thermotherapy helps to relax muscles and increase circulation to the affected area, thus reducing pain and stiffness, although there is some concern that this may, in turn, worsen inflammation and edema. Thermotherapy can be self-applied easily by the patient at home (such as the use of heat packs), and may also be combined with other rehabilitation interventions.^[10]^ However, no systematic review has been performed to evaluate the effectiveness and safety of thermotherapy for shoulder pain. Therefore, the systematic review will assess the efficacy and safety of thermotherapy for shoulder pain.

## 2 Methods

This protocol for this review was developed in accordance with the PRISMA-P guidelines and the Cochrane Handbook. This protocol has been registered on INPLASY (registration number: INPLASY2021110086: https://inplasy.com/inplasy-2021-11-0086/). Ethical approval is unnecessary because this is a literature-based study.

### 2.1 Inclusion criteria for study selection

#### 2.1.1 Types of studies

All randomized controlled trials (RCTs) of thermotherapy for shoulder pain without publication status restriction or writing language. Non-RCTs, quasi-RCTs, uncontrolled trials, reviews, case-controlled studies, animal trials, and laboratory studies will be excluded.

#### 2.1.2 Types of patients

People with shoulder pain regardless of sex, age, or severity and duration of disease.

#### 2.1.3 Types of interventions

Interventions using thermotherapy only were included in this review. Trials that compared thermotherapy with standard treatment and/or placebo were included. Thermotherapy with another active therapy versus the same therapy alone will also be investigated. Trials comparing head to head therapies, such as two different types of diathermy, were not included in this review.

#### 2.1.4 Types of outcomes

The primary outcome of shoulder pain symptom is visual analog scale (0–10), the ability assessment of daily living activities. Adverse events incidence and shoulder range of motion will be accepted as the secondary outcomes.

### 2.2 Search methods for the identification of studies

#### 2.2.1 Electronic searches

Eight databases will be searched from their inception to October 2021. They are as follows: PubMed, Embase, Cochrane Library, ClinicalTrials.gov, China Knowledge Resource Integrated Database (CNKI), Weipu Database for Chinese Technical Periodicals (VIP), Chinese Biomedical Literature Database (CBM), and Wanfang Database. There will be no limitation to study publication status or language. The search terms include shoulder impingement syndrome, rotator cuff, bursitis, adhesive capsulitis, frozen shoulder, shoulder pain, thermotherapy, diathermy, heat therapy, Moxibustion, and RCTs. The equivalent search words will be used in the Chinese databases. The detailed strategies for searching the PubMed database will be presented in Table 1.

**Table 1:** Search strategy used in PubMed.

Search	Search terms
1	((((((shoulder impingement syndrome) OR rotator cuff) OR bursitis) OR adhesive capsulitis) OR frozen shoulder) OR shoulder pain)
2	((((Thermotherapy) OR hyperthermia) OR diathermy) OR heat therapy) OR moxibustion
3	(((random[Text Word] OR randomized[Text Word]) OR control[Text Word]) OR controlment[Text Word]) OR trial[Text Word] AND “humans”[MeSH Terms]
4	#1 AND #2AND #3

#### 2.2.2 Searching other resources

Additionally, the international clinical trials registry platform, dissertation, and gray literature will also be searched to identify systematic reviews related to thermotherapy for shoulder pain. The relevant conference papers, journals will be retrieved manually.

### 2.3 Data collection and analysis

#### 2.3.1 Selection of studies

**Figure 1:**
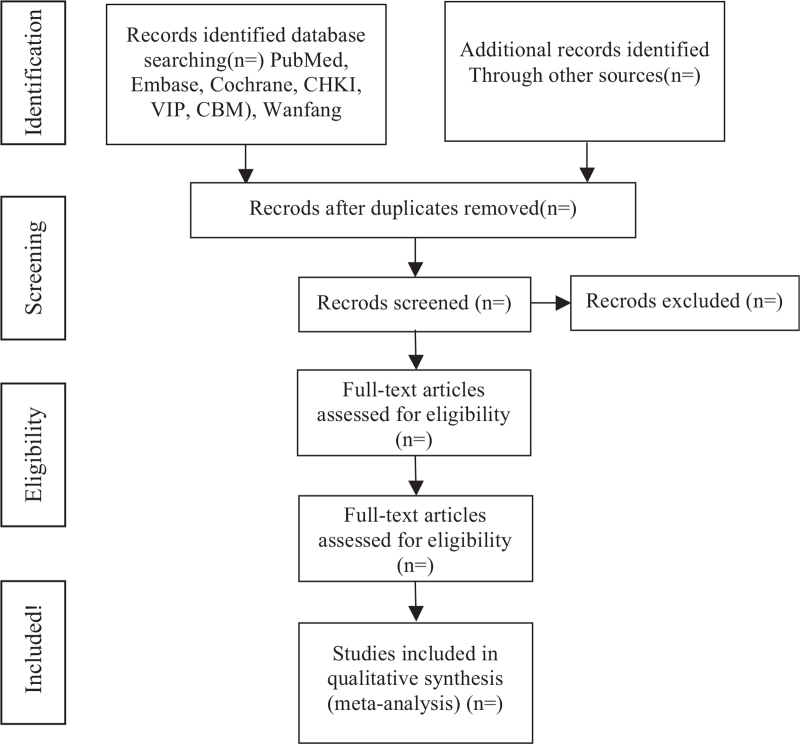
The PRISMA flow diagram of study selection process. PRISMA = preferred reporting items for systematic review and meta-analysis.

For the first version of the review, 2 authors will screen the search yield and identify any potentially eligible citations. We will retrieve the full text of articles judged as being potentially eligible by at least 1 review author. Two review authors will screen the full-text articles for eligibility and resolve any disagreements by discussion or by consulting a third review author.^[11]^ We will record the selection process in sufficient detail to complete a study selection flow diagram (Fig. 1).

#### 2.3.2 Data extraction and management

Before data extraction, a standard form will be prepared for data collection. Two researchers will independently extract data of the included studies and write on the form. Any disagreement will be solved by consensus. The following data will be extracted: the first author, publication year, participants characteristics, interventions, duration of treatment, follow-up, outcome assessment, research results, adverse events, and other detail information. We will contact the original author for complete information when necessary.

#### 2.3.3 Assessment of risk of bias

Two researchers will assess the risk of bias of included studies independently according to the Cochrane collaboration's tool.^[12]^ The tool comprise 7 aspects which are random sequence generation, allocation concealment, the blinding method for patients, researchers and outcomes assessors, incomplete outcome data, and selective reports.^[13]^ Every risk of bias will be classified as low, unclear, and high.

#### 2.3.4 Measures of treatment effect

For continuous data, a mean difference or standardized mean difference with 95% confidence intervals (CIs) will be applied. For dichotomous outcome data, the risk ratio with 95% CIs will be used to evaluate the treatment effect.

#### 2.3.5 Missing data management

If the essential data are not provided, we will try to contact the corresponding author of the articles by email for complete data. If the missing data cannot be obtained, we will analyze the available data.

#### 2.3.6 Assessment of heterogeneity

We will assess clinical and methodological diversity in terms of participants, interventions, outcomes and study characteristics for the included studies, to determine whether a meta-analysis is appropriate.^[14]^ When meta-analysis is appropriate, we will assess and quantify the possible magnitude of inconsistency (i.e., heterogeneity) across studies, using the *I*^2^ statistic with a guide for interpretation as follows: 0% to 40% might not be important; 30% to 60% may represent moderate heterogeneity; 50% to 90% may represent substantial heterogeneity; and 75% to 100% represents considerable heterogeneity. If we identify cases of considerable heterogeneity (defined as *I*^2^ of 75% or greater), we will explore the data further by comparing the characteristics of individual studies and performing subgroup analyses.

#### 2.3.7 Data synthesis

We plan to pool outcomes from trials with similar characteristics (participants, interventions and common comparators, outcome measures, and timing of outcome measurement) to provide estimates of benefit and harm. We plan to synthesize effect estimates using a random-effects meta-analysis model based on the assumption that clinical diversity is likely to exist, and that different studies are estimating different intervention effects. Where we cannot pool data, we plan to present effect estimates and 95% CIs of each trial in tables, and summarize the results in text.

#### 2.3.8 Sensitivity analysis

When there are sufficient studies, sensitivity analysis will be performed to assess the robustness of studies according to methodological quality, sample size, and missing data.

#### 2.3.9 Reporting bias

In order to determine whether outcome reporting bias is present, we will check a priori trial protocols against published reports of trial results (i.e., check if all planned outcomes have results reported). We will compare the fixed-effect estimate against the random-effects model to assess the possible presence of small-sample bias in the published literature (i.e., in which the intervention effect is more beneficial in smaller studies).^[15]^ In the presence of small-sample bias, the random-effects estimate of the intervention is more beneficial than the fixed-effect estimate. If we are able to pool more than 10 trials, we will undertake formal statistical tests to investigate funnel plot asymmetry to detect the possibility of publication bias.

#### 2.3.10 Confidence in cumulative evidence

The quality of evidence will be assessed based on the grading of recommendations assessment, development, and evaluation system, include 4 levels: high, moderate, low, or very low.

## 3 Interpreting results and reaching conclusions

We will follow the guidelines in Chapter 12 of the Cochrane Handbook for Systematic Reviews of Interventions for interpreting results, and will be aware of distinguishing a lack of evidence of eHect from a lack of eHect.^[16]^ We will base our conclusions only on findings from the quantitative or narrative synthesis of included studies for this review. We will avoid making recommendations for practice, and our implications for research will suggest priorities for future research and outline what the remaining uncertainties are in the area.

## Author contributions

**Conceptualization:** Bin Chen, Na Li.

**Data curation:** Lu Wang, Dandan Chen.

**Formal analysis:** Lu Wang, Dandan Chen.

**Funding acquisition:** Bin Chen.

**Guarantor of the review:** Bin Chen, Cimin Shen.

**Investigation:** Cimin Shen.

**Methodology:** Na Li, Lu Wang, Dandan Chen.

**Project administration:** Na Li.

**Resources:** Cimin Shen, Na Li.

**Software:** Lu Wang, MD, Dandan Chen.

**Supervision:** Cimin Shen, Na Li.

**Validation:** Bin Chen, Cimin Shen.

**Visualization:** Na Li, Lu Wang, Dandan Chen.

**Writing – original draft:** Bin Chen, Cimin Shen.

**Writing – review & editing:** Bin Chen, Cimin Shen, Na Li.
